# Supplementary material for: Detection of Tobacco Bacterial Wilt Caused by Ralstonia solanacearum by Combining Polymerase Chain Reaction with an α-Hemolysin Nanopore
Source: Nanomaterials (Basel). 2023 Jan 13;13(2):332. doi: 10.3390/nano13020332 (PMC9863824; doi:10.3390/nano13020332)
Supplement: Supplementary file 1 [file nanomaterials-13-00332-s001.zip › nanomaterials-2064503-supplementary.pdf]

Supplementary materials

Detection of tobacco bacterial wilt caused by *Ralstonia solanacearum* by combining polymerase chain reaction with α-hemolysin nanopore

Ying Wang[1], Yusen Li[1], Xin Zhou[1], Wenna Zhang[2], Shusheng Zhang[1], Dongmei Xi[1,*]

[1] College of Life Science, Shandong Provincial Key Laboratory of Detection Technology for Tumor Markers, Linyi University, Linyi 276005, P. R. China
[2] Shandong (Linyi) Institute of Modern Agriculture, Zhejiang University, Linyi 276000, China.
* Correspondence: dongmxi@126.com

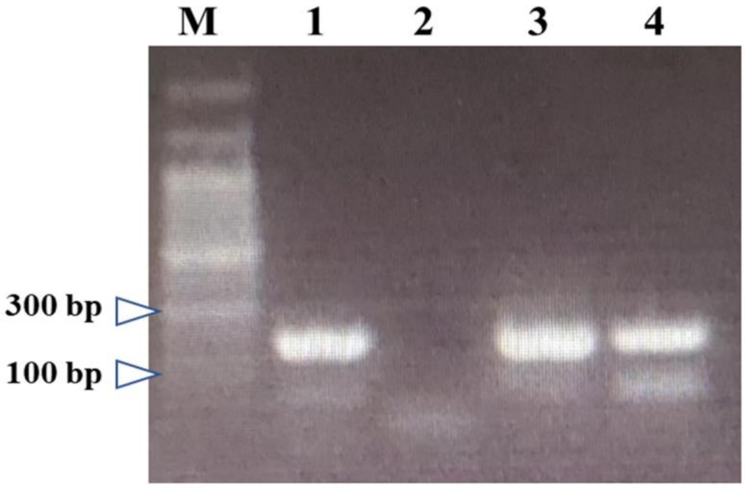

Figure S1. 2% Agarose gel electrophoresis of the PCR product. Lane 1, DNA Ladder L(100~3000bp). Lane 2, PCR product of pure water (negative control). PCR product of solution in *cis* chamber (lane 3) and *trans* chamber (lane 4). Strand A in the two chambers were purified by the Universal DNA Purification kit, which was used as the template for PCR.

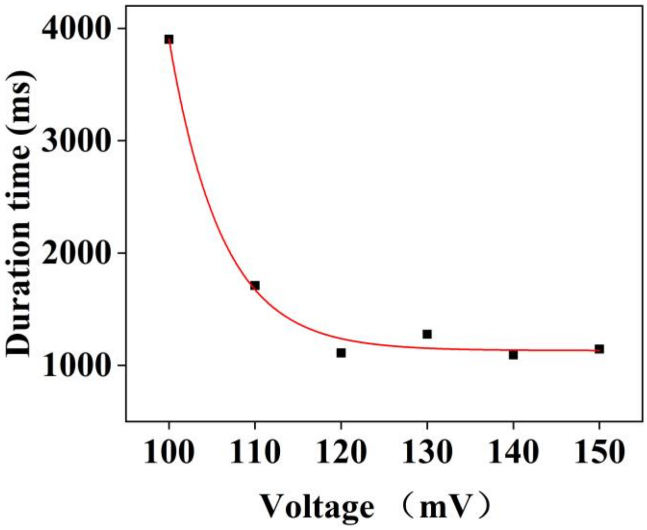

Figure S2. Durations of the synthetic strand A were decreased as the voltage increased from 100 to 150 mV.

To determine whether strand A was translocated, a test of the relationship between duration and voltage was performed. Strand A was systhsized and purified by Takara Bio Inc. The results show duration of strand A was decreased from 3902.64 ms to 1145.51 ms as the voltage increased from + 100 to + 150 mV. At the end of the translocation test, the solution in the two chambers were purified by the Universal DNA Purification kit, which were used as the templates for PCR. The results show that the 198 nt fragments were observed on a 2% agarose gel. These experimental results verified that strand A was translocated to the *trans* chamber.

Duration of the unpurified lambda exonuclease-treated PCR products containing strand A ranged from 20.11 ms to 7232.85 ms at + 100 mV voltage (Figure 3). However, duration of the synthesized strand A was between 52.03ms-35364.35 ms at + 100 mV voltage. We speculated that different buffer solution of purified and unpurified strand A may lead to different higher structures, and thus different durations. In addition, strand A may interact with other ions in the lambda exonuclease- PCR buffer or TE buffer, which cause different non-covalent interactions between strand A and nanopores, including electrostatic interactions, π-effect, and hydrophobic interactions.

In addition, the lambda exonuclease-treated PCR products were purified by the Universal DNA Purification Kit to obtain relatively pure strand A. The voltage results show duration of strand A was decreased from 4545.95 ms to 518.84 ms as the voltage increased from + 100 mV to + 130 mV, and duration of the purified strand A was between 39.8 ms-101000 ms at + 100 mV. Duration of the purified lambda exonuclease-treated PCR product was similar to that of the synthetic strand A, which was significantly longer than that of the unpurified lambda exonuclease-treated PCR products.

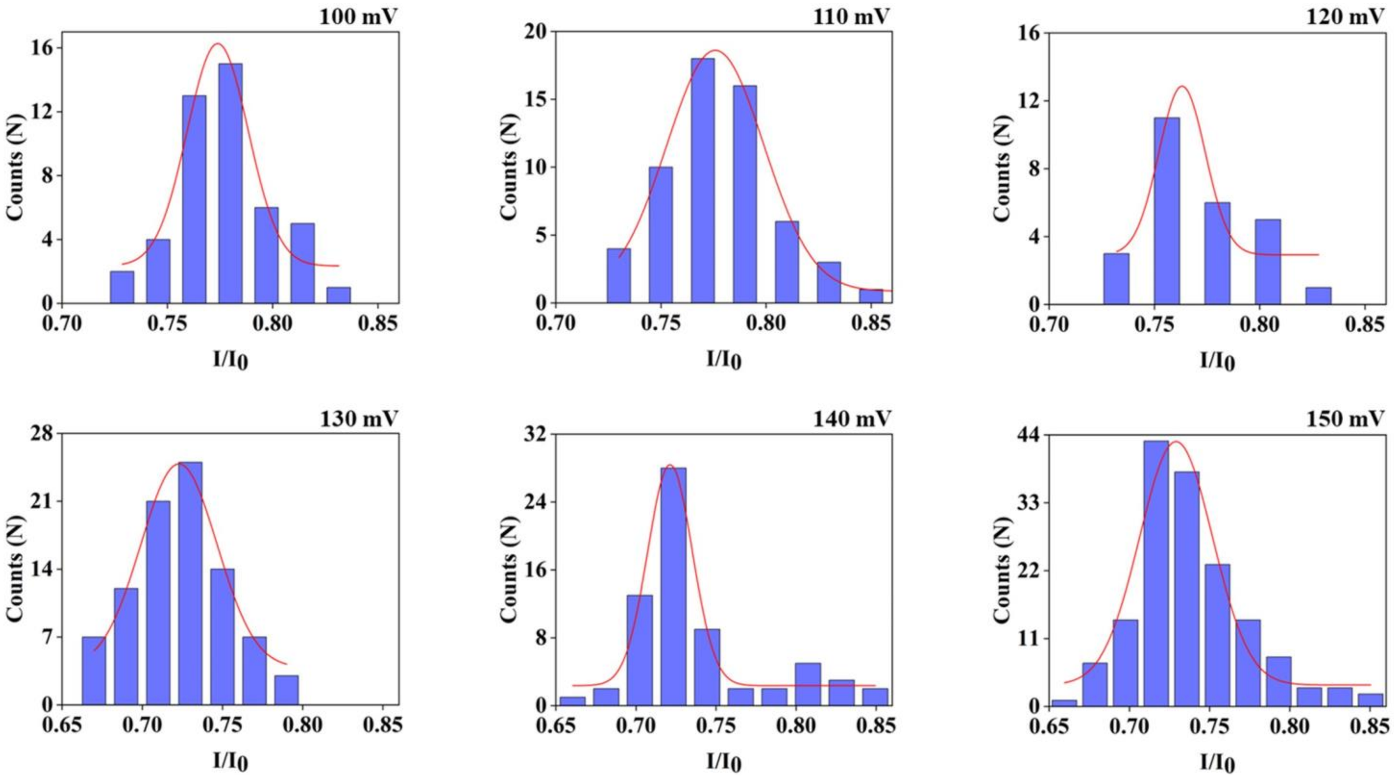

Figure S3. I/I_0 histogram of events of the strand A from +100 mV to +150 mV in 0.75 M/3 M (*cis*/*trans*) KCl.

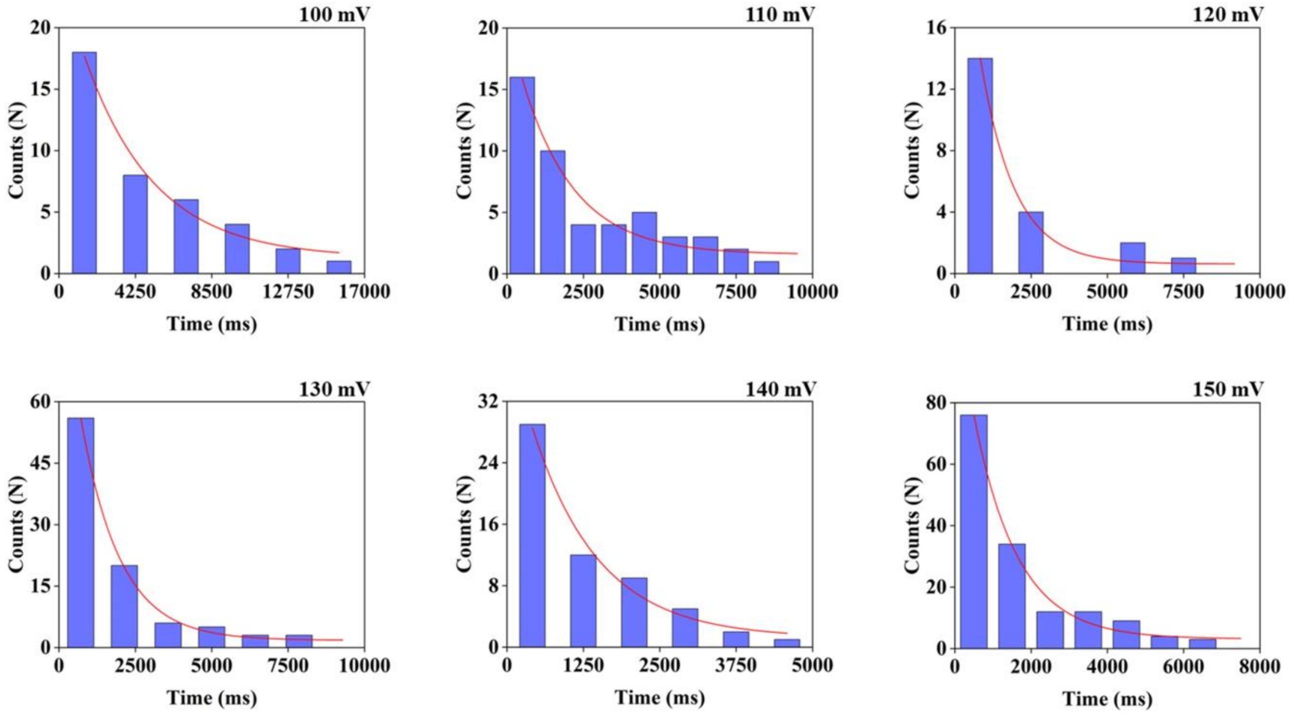

Figure S4. Duration histogram of events generated by strand A from +100 mV to +150 mV in 0.75 M/3 M (*cis*/*trans*) KCl.

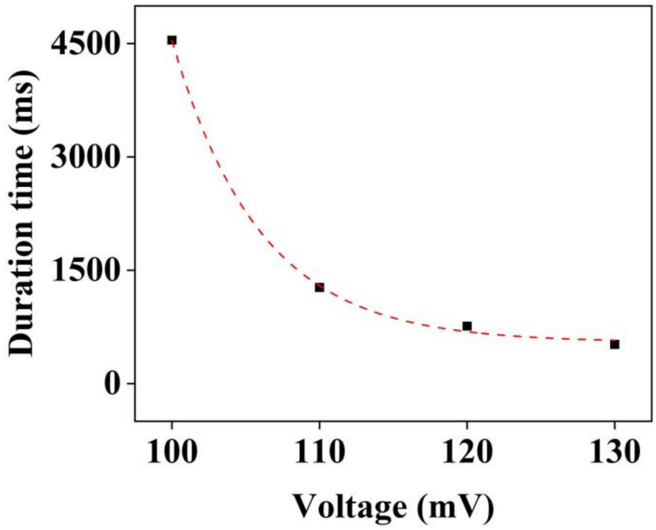

Figure S5. Durations of the Universal DNA Purification kit-purified strand A were decreased as the voltage increased from 100 to 130 mV.

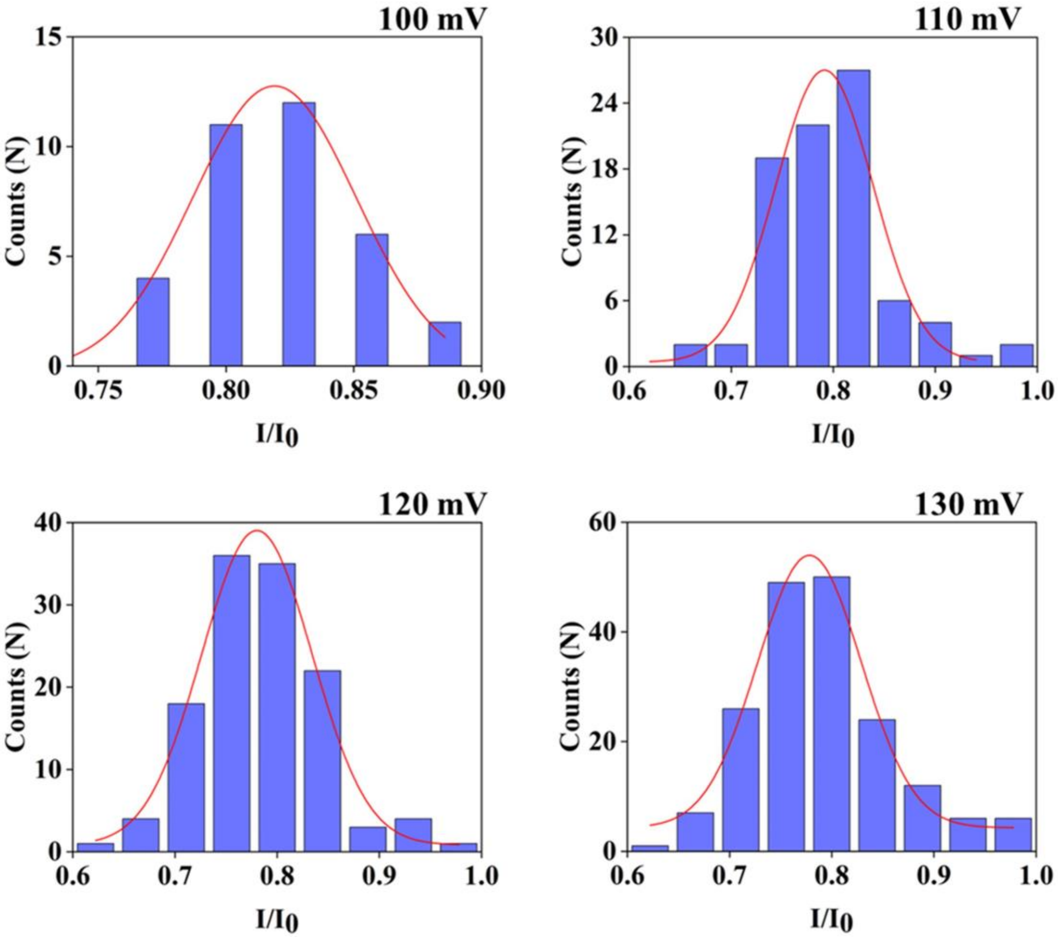

Figure S6. I/I_0 histogram of events generated by the Universal DNA Purification kit-purified strand A under gradient voltage from +100 mV to +130 mV in 0.75 M/3 M (*cis*/*trans*) KCl.

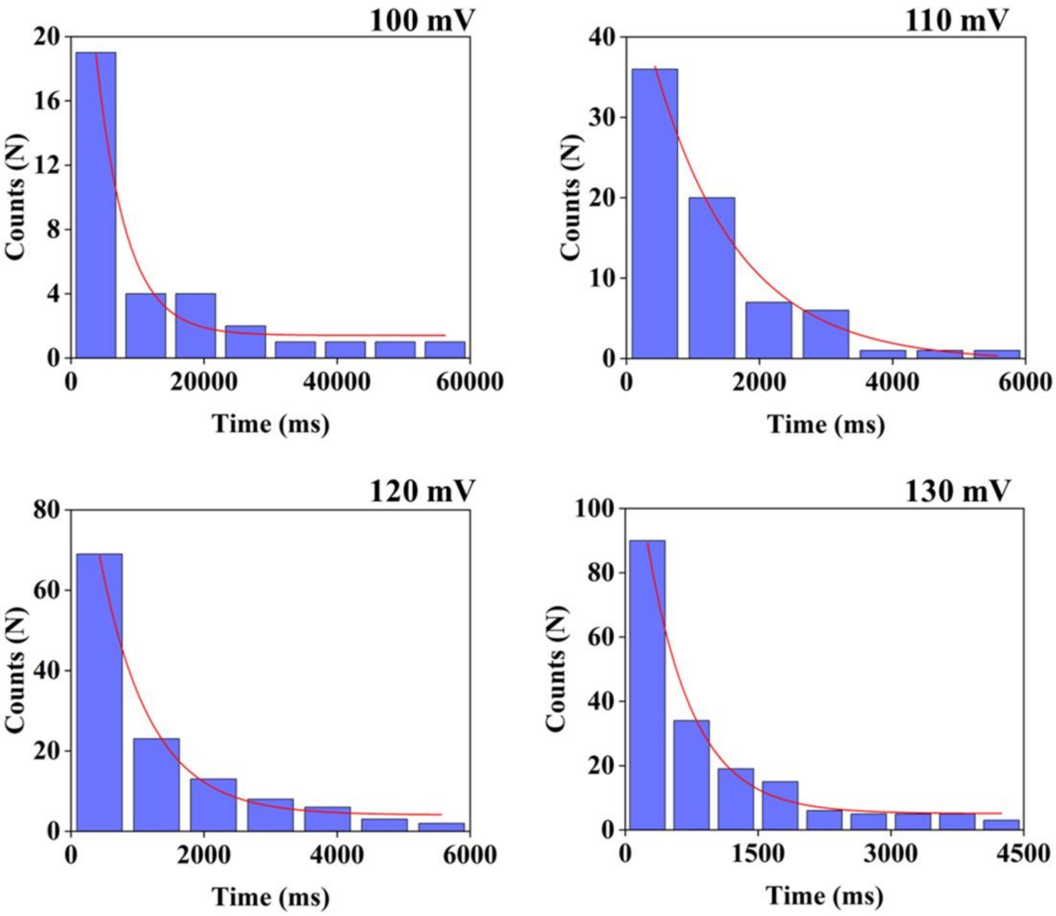

Figure S7. Duration histogram of events generated by the Universal DNA Purification kit-purified strand A under voltage from +100 mV to +130 mV in 0.75 M/3 M (*cis*/*trans*) KCl.

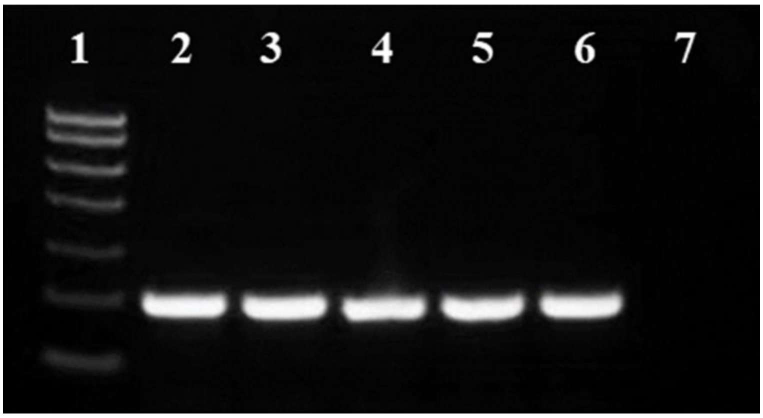

Figure S8. 2% Agarose gel electrophoresis of the PCR product. Lane 1, *Trans* DNA Marker I. Lane 2, PCR product of *R. solanacearum* genome. Lane 3-7, PCR product of field tabacco samples of 1-5.

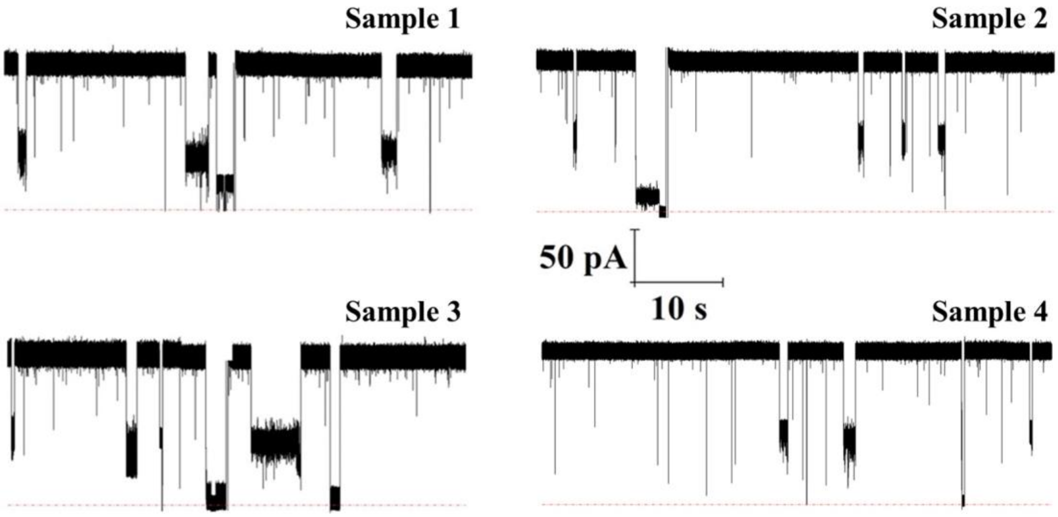

Figure S9. Current trace of the 4 positive field samples. All nanopore data were recorded at +100 mV in 0.75 M/3 M (*cis/trans*) KCl, and the same below.

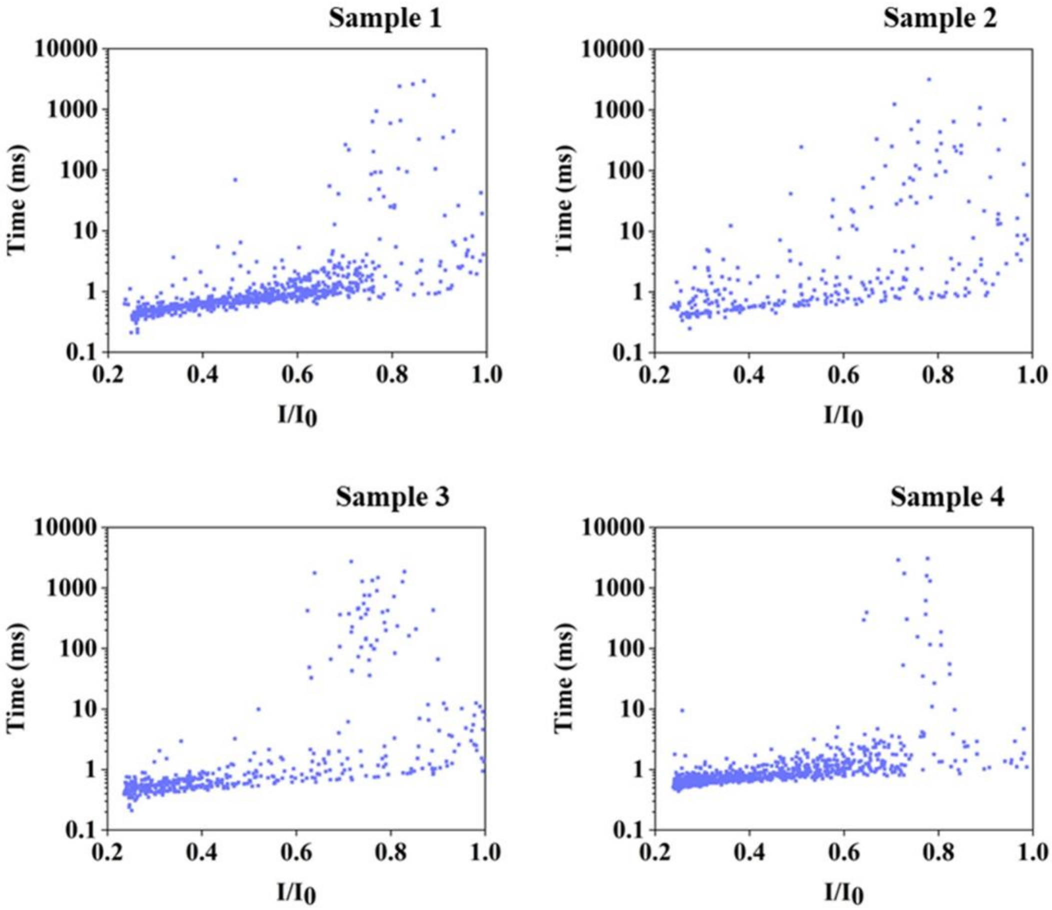

Figure S10. Scatter plot of the 4 positive field samples.

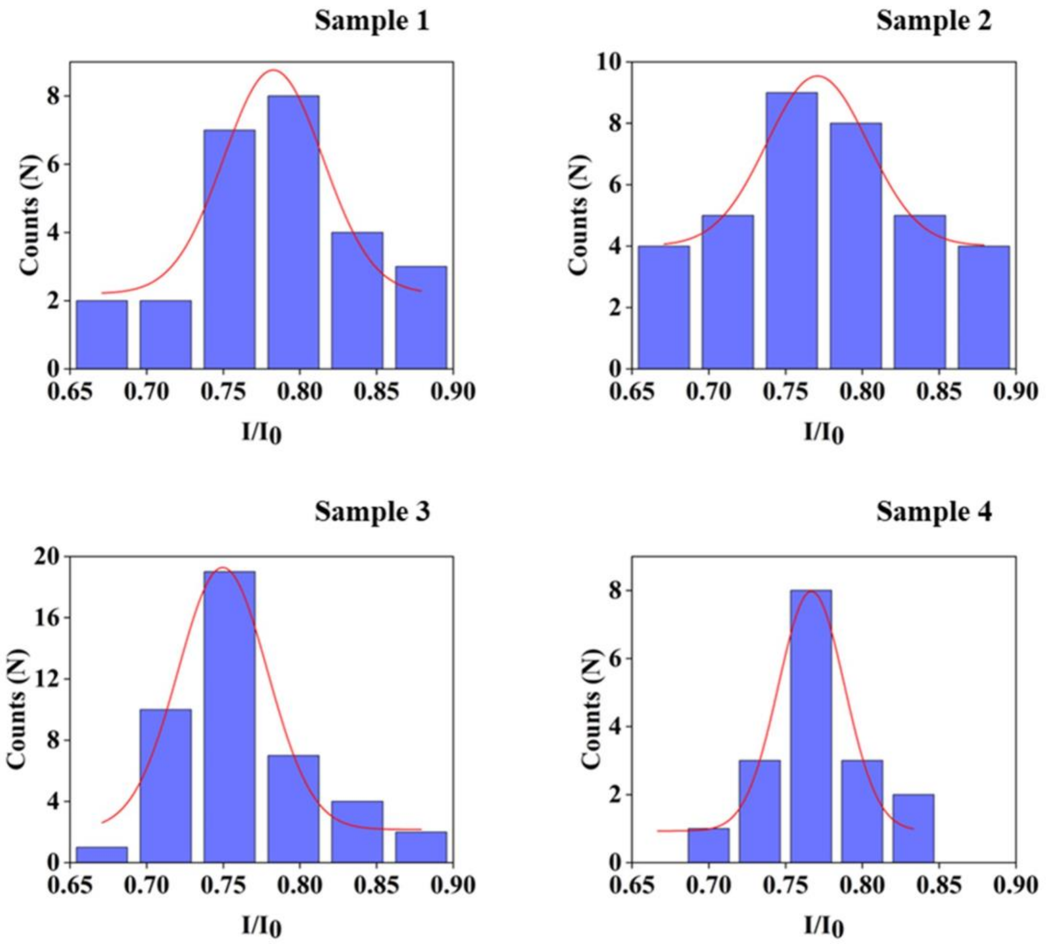

Figure S11. I/I_0 histogram of the events produced by the 4 positive field samples.

Field sample 1

Download GenBank Graphics

Ralstonia solanacearum strain FJAT1303.F1 chromosome, complete genome

Sequence ID: CP052128.1 Length: 3649900 Number of Matches: 1

Range 1: 1185402 to 1185566 GenBank Graphics ▼ Next Match ▲ Previous Match

Score	Expect	Identities	Gaps	Strand
287 bits(155)	4e-73	162/165(98%)	2/165(1%)	Plus/Minus

```
Query  3        ATC-GCGTCCAGGCGACCTACATCGACGAACGAACGAAGTGGCGAACGGGCTTTCCCAAC  61
                ||| ||| ||||||||||||||||||||||||||||||||||||||||||||||||||||
Sbjct  1185566  ATCAGCGCCCAGGCGACCTACATCGACGAACGAACGAAGTGGCGAACGGGCTTTCCCAAC  1185507

Query  62       GGAAGCAATGACAACCCCACGTCCAAGCTGCGCTCTTTCAGGGCAAAGGCAACCTACGTG  121
                ||||||||||||||||||||||||||||||||||||||||||||||||||||||||||||
Sbjct  1185506  GGAAGCAATGACAACCCCACGTCCAAGCTGCGCTCTTTCAGGGCAAAGGCAACCTACGTG  1185447

Query  122      TACGACCACACCTACGGCGTGACCGCTGGCGT-TTCAAGGTGACC  165
                |||||||||||||||||||||||||||||||| ||||||||||||
Sbjct  1185446  TACGACCACACCTACGGCGTGACCGCTGGCGTCTTCAAGGTGACC  1185402
```

Field sample 2

Download GenBank Graphics

Ralstonia solanacearum strain FJAT1303.F1 chromosome, complete genome

Sequence ID: CP052128.1 Length: 3649900 Number of Matches: 1

Range 1: 1185402 to 1185562 GenBank Graphics ▼ Next Match ▲ Previous Match

Score	Expect	Identities	Gaps	Strand
285 bits(154)	1e-72	159/161(99%)	1/161(0%)	Plus/Minus

```
Query  3        GCGTCCAGGCGACCTACATCGACGAACGAACGAAGTGGCGAACGGGCTTTCCCAACGGAA  62
                ||| ||||||||||||||||||||||||||||||||||||||||||||||||||||||||
Sbjct  1185562  GCGCCCAGGCGACCTACATCGACGAACGAACGAAGTGGCGAACGGGCTTTCCCAACGGAA  1185503

Query  63       GCAATGACAACCCCACGTCCAAGCTGCGCTCTTTCAGGGCAAAGGCAACCTACGTGTACG  122
                ||||||||||||||||||||||||||||||||||||||||||||||||||||||||||||
Sbjct  1185502  GCAATGACAACCCCACGTCCAAGCTGCGCTCTTTCAGGGCAAAGGCAACCTACGTGTACG  1185443

Query  123      ACCACACCTACGGCGTGACCGCTGGCGT-TTCAAGGTGACC  162
                |||||||||||||||||||||||||||| ||||||||||||
Sbjct  1185442  ACCACACCTACGGCGTGACCGCTGGCGTCTTCAAGGTGACC  1185402
```

Field sample 3

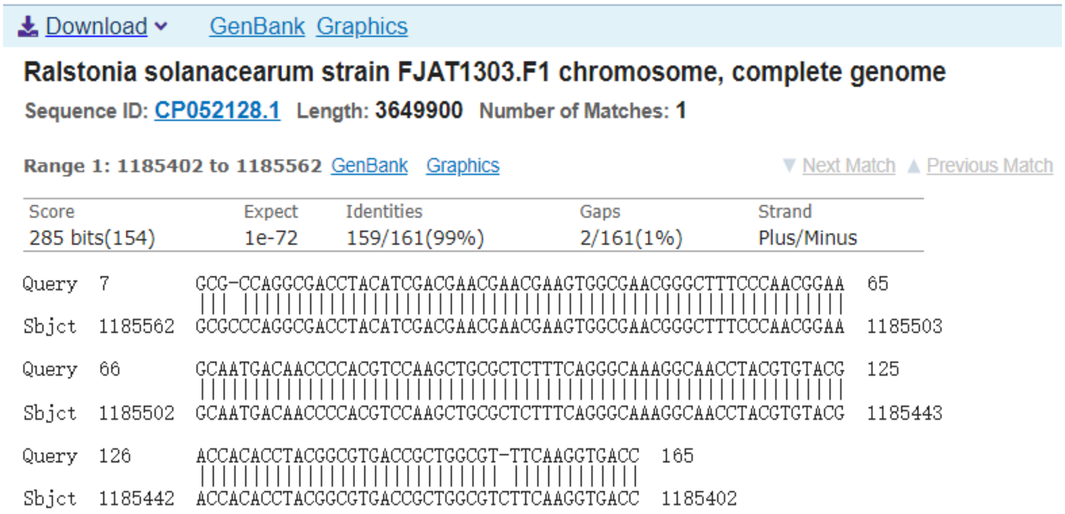

Field sample 4

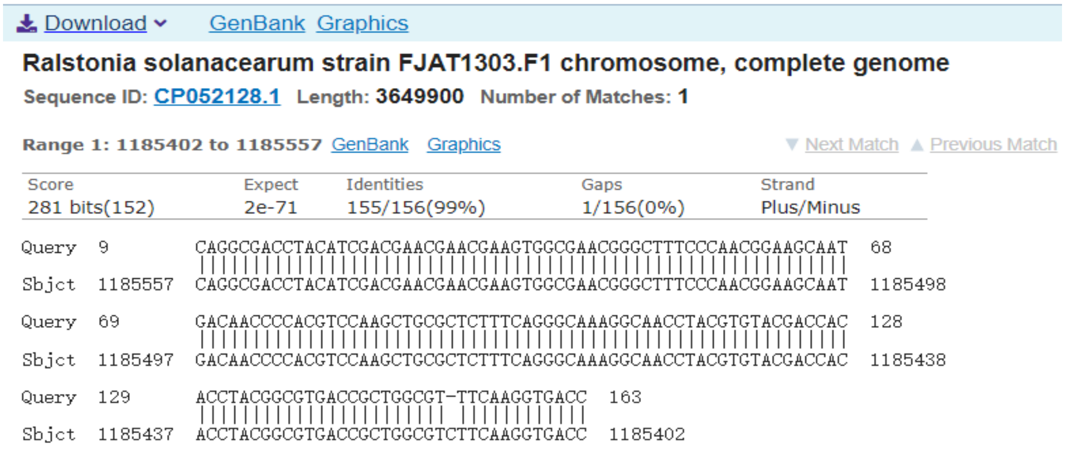

Figure S12. DNA sequencing of the 4 positive field sample. Alignment of DNA sequencing results of the 4 positive field samples by Basic Local Alignment Search Tool in NCBI website.

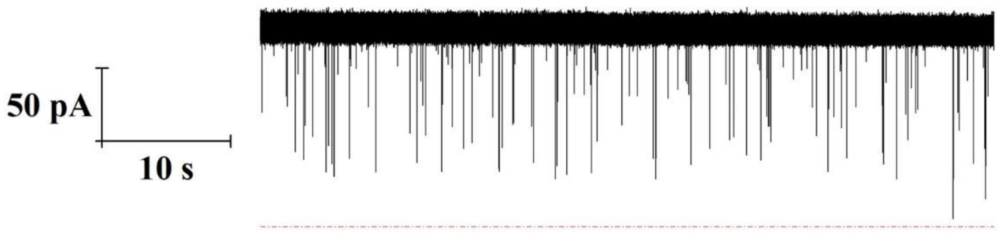

Figure S13. Current trace of the negative field sample (sample 5).

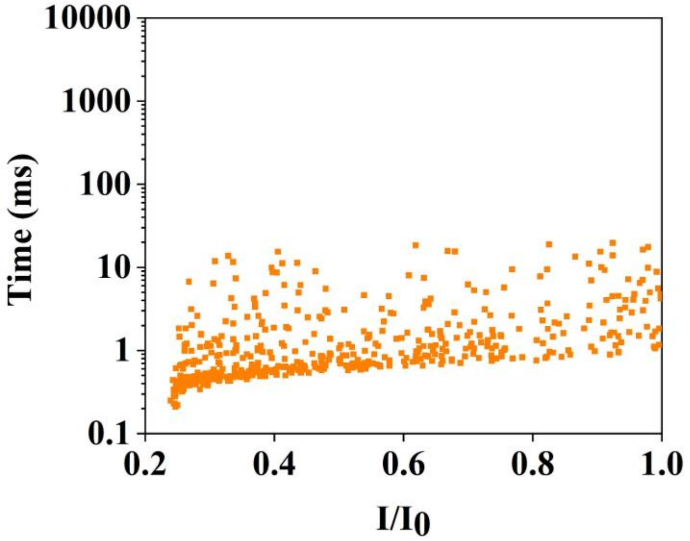

Figure S14. Scatter plot of the sample 5 (n=524).
